# Impact of Medical Student Disciplinary Actions on the United States National Resident Match

**DOI:** 10.7759/cureus.24583

**Published:** 2022-04-29

**Authors:** Malcolm D Mattes, Norman D Ferrari III

**Affiliations:** 1 Radiation Oncology, Rutgers Cancer Institute of New Jersey, New Brunswick, USA; 2 Medical Education, West Virginia University School of Medicine, Morgantown, USA

**Keywords:** adverse actions, probation, medical residency, medical education, undergraduate medical education

## Abstract

Introduction: Each year, the United States National Resident Matching Program describes the relative importance of a number of factors in the residency match for each speciality. However, the impact of disciplinary actions taken by a school when a student fails to meet certain expectations is not specifically evaluated but may have a major impact on a physician’s future performance.

Methods: This study used electronic surveys sent to deans of medical education and residency program directors (PDs) to assess the way disciplinary actions are used at US allopathic medical schools, and the perceived implications of those actions on the residency match.

Results: Thirty-three deans and 158 PDs participated (response rates of 26% and 22%, respectively). The median percentage of students put on probation each year as a function of class size was 3.3% (interquartile range [IQR] 2% to 6%). Three institutions reported putting greater than 10% of their students on probation each year and one institution reported putting 22% of their students on probation each year. A student's risk of failing to match was thought to be very or extremely likely (to deans and PDs, respectively) if there was a history of failed coursework (18.8% and 41.2%, *p *= 0.017), academic probation (34.4% and 67.1%, *p *= 0.009), or professionalism probation (78.1% and 83.9%, *p *= 0.016). The differences between each of the above types of disciplinary action’s impact on the likelihood of interviewing (*p *< 0.001) and risk of failure to match (*p *< 0.001) were also significant among both groups.

Conclusion: Significant variability exists in the use and reporting of disciplinary actions at US medical schools. A history of these adverse actions, even if successfully remediated, was thought to negatively impact a student’s likelihood to interview and match. Greater standardization in the use and reporting of disciplinary actions would be appropriate to ensure equitable treatment of students nationwide.

## Introduction

In all aspects of education, it is important to ensure that graduating students achieve the academic and professional competencies that are expected of them. Should a student fail to meet these expectations, some disciplinary action and remediation may be appropriate to help that student get back on track. This is particularly important in medical school, where at its most basic level, the goal of medical educators is to produce a group of high-quality physicians to serve the public, and also to protect the public from those who will not be. Organizations like the Association of American Medical Colleges (AAMC) and the Accreditation Council for Graduate Medical Education (ACGME) have described core competencies that medical students and physicians should meet [1-2], but for good reason, there is flexibility in how these competencies are achieved. As long as the caliber of students entering different schools varies, the optimal means of educating those students, including the rigor of examinations/grading and the extent of use of disciplinary actions, should also vary to some degree.

Hopefully, a school’s disciplinary actions should most often result in the desired outcome of successful remediation and eventual graduation. However, there is relatively little medical literature addressing how a student’s history of disciplinary actions (even if successfully remediated) may impact that student’s ability to obtain interviews and match in their specialty of choice. For instance, the impact of disciplinary/adverse actions is not specifically evaluated in the National Resident Matching Program (NRMP) Charting Outcomes in the Match or the Results of Program Director Survey [3-4]. Information on adverse actions imposed on the student by the medical school is supposed to be contained in the Medical Student Performance Evaluation (MSPE) [5]. However, previous studies have demonstrated that this might not always be the case [6-7]. Having a better understanding of how disciplinary actions are used at medical schools, and the implications of those disciplinary actions on the residency match would be beneficial for students as they choose a medical school and understand the consequences of failing to meet expectations as they progress through their education. Furthermore, a better understanding of how disciplinary actions are used at medical schools could also be beneficial to administrative leaders in medical schools as they develop institutional policies for disciplinary actions that maximize their students’ ultimate potential for success.

The purpose of this study is to better characterize how disciplinary actions are utilized at United States (US) medical schools, as assessed by leaders in medical student and resident education. We will also assess the perceived impact of a student having a personal history of different types of disciplinary actions on their ability to successfully match into their preferred medical specialty.

## Materials and methods

This study involved two separate electronic surveys: one 18-item survey designed for deans of medical education at US allopathic medical schools, and a separate 10-item survey designed for program directors (PDs) of US residency programs affiliated with an allopathic medical school in the fields of internal medicine, general surgery, family medicine, obstetrics/gynecology, pediatrics, neurology, and radiology. These particular specialties were included to cover a variety of medical and surgical specialties, and because most medical students also rotate through these specialties during their clinical clerkships. Osteopathic medical schools were excluded due to differences in their curricular content and approach to implementation compared to allopathic schools. Research Electronic Data Capture (REDCap) (Vanderbilt University, Tennessee, USA), a secure web application used to build and manage online surveys and databases, was used to develop and disseminate the surveys [8]. Only those deans and PDs whose email addresses were freely accessible online via school websites were included. In total, invitations to participate were sent to 126 deans and 711 PDs. This study was granted an exemption determination by West Virginia University Institutional Review Board (No. 1608247204). Participation was anonymous, completely voluntary, and no financial incentive was provided. Up to three reminder emails were sent to non-responders. All responses were received between August and November 2019.

The question development for both the dean and PD surveys followed the CHEcklist for Reporting Results of Internet E-Surveys (CHERRIES) criteria [9]. Question structures were predominately multiple-choice and 5-point Likert-type scales, though free-text comment boxes were also available where indicated. Deans were asked about the types of disciplinary actions used at their medical school, as well as the frequency and duration of their use. They were asked about the degree of variability that they believed existed at different US medical schools in the criteria for probation, and the number of students put on probation. Finally, deans were asked to rate failed coursework, academic probation, and professionalism probation in terms of how likely (ranging from (1) not at all, to (5) extremely) they believe that successful remediation of each of these disciplinary actions is to impact a residency PDs decision to interview the student or a student’s risk of failing to match into a residency program. The survey did not specifically ask about other factors that are of significance in the residency match (e.g. United States Medical Licensing Examination [USMLE] scores, grades, research, letters of recommendation, personal statement, etc.) because these other factors are already reported upon in the NRMP Program Director Survey. However, participants were asked to consider the relative importance of these other factors compared to the importance of the disciplinary action when responding. The PDs were asked a similarly structured set of questions applicable to them. The complete survey questions are shown in Appendix A.

Descriptive statistics were used to summarize the findings of both surveys. Likert-type scales were treated as ordinal variables, and the Mann-Whitney U test, Spearman’s rank correlation, Kruskal-Wallis H test, and Friedman test were used where appropriate. Post hoc analyses involved Wilcoxon signed-rank tests with a Bonferroni correction. Statistical analysis was performed using Statistical Package for Social Sciences (SPSS) version 20.0 (IBM Corporation, Armonk, NY). Unless otherwise noted, a p-value < 0.05 was considered statistically significant.

## Results

A total of 33 deans of medical education (response rate 26.2%; 14.7% margin of error at 95% confidence interval) and 158 PDs (response rate 22.2%; 6.9% margin of error at 95% confidence interval) replied to the survey. The average age of the deans was 45.6 (standard deviation [SD] 8.5) years and of the PDs was 49.4 (SD 8.8) years. Additional characteristics of the participants can be found in Table 1.

**Table 1:** Characteristics of medical school dean of education and residency program director (PD) participants

Deans		Program Directors	
Characteristic	Number (%)	Characteristic	Number (%)
Gender		Gender	
Female	11 (33.3%)	Female	73 (46.2%)
Male	22 (66.6%)	Male	85 (53.8%)
Type of School		Residency Program	
Public	23 (69.7%)	Obstetrics/Gynecology	31 (19.6%)
Private	10 (30.3%)	Pediatrics	26 (16.5%)
Class Size		Internal Medicine	25 (15.8%)
< 100 students	4 (12.1%)	Surgery	21 (13.3%)
101 – 150 students	14 (42.4%)	Neurology	20 (12.6%)
151 – 200 students	7 (21.2%)	Family Medicine	18 (11.4%)
201 – 250 students	6 (18.2%)	Radiology	14 (8.9%)
> 250 students	2 (6.1%)	Other/Unknown	3 (1.9%)
Number of Years as Dean		Number of Years as PD	
1 – 5 years	14 (42.4%)	1 – 5 years	84 (53.2%)
6 – 10 years	7 (21.2%)	6 – 10 years	35 (22.1%)
> 10 years	11 (33.3%)	> 10 years	39 (24.7%)
Unknown	1 (3.0%)		

When a medical student fails to meet academic and/or professional standards, 79% of deans reported the use of probation as a potential disciplinary action at their medical school. Among these, 68% distinguish between academic and professionalism probation, and 42% reported the use of “other” disciplinary actions besides probation or suspension, for instance, “warning” or “incomplete.” One dean commented that failures in professionalism were more likely to lead to “probation” at that dean’s school, whereas academic failures were typically labeled “incomplete for remediation.” Individualized/focused remediation of disciplinary actions consisted of a variety of activities inclusive of but not limited to counseling, additional assignments, repeating coursework, leave of absence, or dismissal, depending on the circumstances. 

Among those schools that utilized probation, the median reported duration of probation was 10.5 months (IQR six to 12 months) and the median percentage of students put on probation each year as a function of class size was 3.3% (IQR 2% to 6%). Three institutions reported putting greater than 10% of their students on probation each year and one institution reported putting 22% of their students on probation each year. Seventy-two percent of deans felt that the number of students they put on probation each year was “about average” compared to other medical schools, including 83% of the deans at schools that were in the upper quartile for probation use. A Spearman’s rank-order correlation was used to determine the relationship between the percentage of students each school put on probation each year and how the dean felt this number compared to that at other schools. There was a non-significant trend toward a correlation between the two (r_s_ = 0.340, p = 0.104). Residency PDs also felt that there was moderate variability (median Likert-type score 3 [IQR 2 to 4]) in the number of students put on probation at different US medical schools, as well as the criteria for probation (median Likert-type score 3 [IQR 2 to 4]).

**Figure 1:**
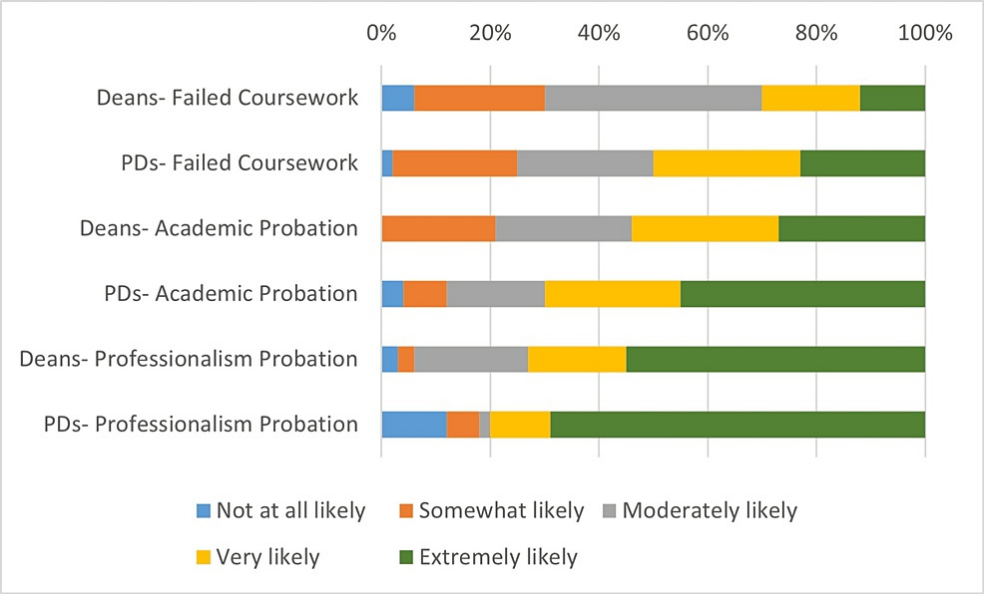
Deans and program directors (PDs) rated the perceived impact of successful remediation of failed coursework, academic probation, and professionalism probation on a residency PDs decision to interview the student

**Figure 2:**
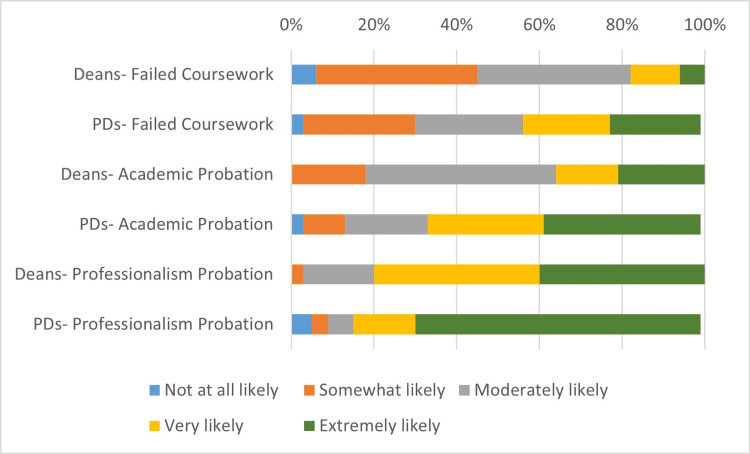
Deans and program directors (PDs) rated the perceived impact of successful remediation of failed coursework, academic probation, and professionalism probation on a student’s risk of failing to match into a residency program

Figures 1, 2, and Table 2 summarize the deans' and PDs' views of the perceived impact of successful remediation of failed coursework, academic probation, and professionalism probation on a residency program director's decision to interview the student, and a student's risk of failing to match into a residency program. For both deans and PDs, a history of professionalism probation was thought to be significantly more likely than academic probation and failed coursework to make an impact on the likelihood of interview and failure to match (p < 0.008). The PDs were significantly more likely than the deans to believe that failed coursework (p = 0.017), academic probation (p = 0.009), and professionalism probation (p = 0.016) were each associated with a higher likelihood of a student’s failure to match. However, there were no statistically significant differences in the views of the deans and PDs in regards to the effect of each disciplinary action on the likelihood of interviewing. Of note, the medical specialty of the PDs did not significantly impact any of the responses to this set of questions.

**Table 2:** Percentage of deans and program directors who thought it very or extremely likely (Likert-type score 4-5) that a history of failed coursework, academic probation, or professionalism probation would impact a residency program director's decision to interview the student, and a student's risk of failing to match into a residency program

	Deans			Program Directors		
	Failed Coursework	Academic Probation	Professionalism Probation	Failed Coursework	Academic Probation	Professionalism Probation
Decision to interview	31.3%	53.1%	71.9%	50.0%	69.6%	80.4%
Failure to Match	18.8%	34.4%	78.1%	41.2%	67.1%	83.9%

## Discussion

In this study, we have characterized the variability that exists in the use of disciplinary actions across US medical schools. Most medical school deans of education and residency program directors appeared to be somewhat aware of this variability, though there was more of a perception among PDs that a history of adverse actions, even if successfully remediated, may negatively impact a student’s likelihood to interview and match. Interestingly, these findings appeared to be relatively independent of the PDs’ specialty. Of course, a critical component of this discussion is not only how these disciplinary actions are implemented but how they are reported to residency programs in the MSPE. The AAMC has recently made recommendations to improve the MSPE with more consistent content and terminology in an attempt to improve the level of standardization and transparency and ensure fairness [5]. However, these recommendations do not change the fact that institutions may use progressive disciplinary actions with internal designations that may be omitted from the MSPE if the student successfully remediates them. Given the perceived implications that reporting adverse disciplinary actions to residency programs appeared to have in this study, more effort at standardizing the terminology that schools use to describe these actions seems appropriate to ensure that schools that formally discipline a high percentage of students are not hurting their students’ chances of matching any more than schools that formally discipline far fewer.

Interestingly, the type of disciplinary action also had a clear impact on participants, with professionalism issues viewed more negatively than academic deficiencies. This may be because what constitutes a violation of professionalism may be more heterogeneous and subjective than academic violations, thus making them harder to interpret, or alternatively that problems with professionalism may be more difficult to remediate [10]. Several studies have reported a correlation between unprofessional behavior during medical school and the probability of a student becoming a "problem resident," or for state licensing boards to take disciplinary action against an individual as a practicing physician [11-13].

The major limitation of this study is that it measured perceptions rather than outcomes, and we do not know based on our findings or the available medical literature whether this population of students with a history of adverse actions on their MSPE fails to match, obtain a position in the post-match Supplemental Offer and Acceptance Program (SOAP), or obtain any Graduate Medical Education (GME) position at all, at a higher frequency than other similar students. Another important limitation is self-selection bias, in which those who chose to respond may not be representative of the entire population of deans and PDs in the United States. Despite our relatively low response rate, the variety of schools, specialties, and years of experience encompassed by our participants lead us to believe that our findings should be relatively generalizable. Another potential source of bias is the inconsistent nomenclature used for disciplinary actions; we tried to account for this by allowing participants to write in a free text format any variations at their institution from commonly used terms like probation and suspension. The recollections of the deans of the prevalence of probation at their school are also subject to cognitive biases, including those related to memory, desire to conform to perceived norms, and recent experiences. We also did not collect any data on whether views on failed coursework differ for a preclinical course versus a clinical clerkship, the threshold for pass/fail at each school, whether prior personal experience working with problem students or residents may have impacted the response, or whether PDs even consistently look at the MSPE in their decision-making process. Despite these limitations, our findings do provide an overview of the use of disciplinary actions at US medical schools, which students and administrators alike may find valuable in building a productive learning environment.

## Conclusions

This study describes the variability that exists in the use of disciplinary actions across US medical schools, as well as potentially inconsistent reporting of these adverse actions on the MSPE. Our findings also suggest a perception, particularly among residency PDs that a history of these adverse actions, even if successfully remediated, may negatively impact a student’s likelihood to interview and match. We recommend greater standardization in the reporting of disciplinary actions so that students from schools with a more rigorous disciplinary system may still be evaluated as equitably as any other students in the residency match.

## Appendices

Appendix A

**Table 3:** Survey questions for deans of medical education MSPE: Medical Student Performance Evaluation

QUESTION	ANSWER CHOICES
1 (multiple choice). When a medical student fails to meet academic and/or professional requirements/standards, what consequences does your medical school employ? (1a) Probation (1b) Suspension (1c) Dismissal (1d) Other	Yes/No
2 (multiple choice). Do you differentiate between academic and professionalism probation?	Yes/No/Other
3 (written value). What is the most common duration of probation?	Answers ranged from two months to greater than one year
4 (multiple choice). Approximately how many medical students at your institution (at all levels of training) are put on probation each year?	0 to 5 Students, 6 to 10 Students, 11 to 15 Students, 16 to 20 Students, 26 to 30 Students
5 (Likert scale). How do you think the number of students your school puts on probation compares to other U.S. medical schools?	(1) Much less than average (2) Somewhat less than average (3) About average (4) Somewhat more than average (5) Much more than average
6 (written values). What is the most common duration of suspension?	Answers ranged from one month to 12 months
7 (multiple choice). Approximately how many medical students at your institution (at all levels of training) are put on suspension each year?	0 to 3 students, 4 to 6 students, 10 to 12 students
8 (Likert scale). How do you think the number of students your school puts on suspension compares to other U.S. medical schools?	(1) Much less than average (2) Somewhat less than average (3) About average (4) Somewhat more than average (5) Much more than average
9 (Likert scale). How much variability in the number of students put on probation or suspension as disciplinary actions do you believe exists at different U.S. medical schools?	(1) Not at all variable (2) Somewhat variable (3) Moderately variable (4) Very variable (5) Extremely variable Unsure
10 (Likert scale). How much variability in the criteria to put students on probation or suspension as disciplinary actions do you believe exists at different U.S. medical schools?	(1) Not at all variable (2) Somewhat variable (3) Moderately variable (4) Very variable (5) Extremely variable
11 (multiple choice). Does your institution always include a record of probation or suspension in the MSPE/Dean's Letter?	Yes/No/Unsure
12 (specify). If they answered no or unsure to *Q11, they were asked to please specify:	Answers varied to include the independent practices of some schools
13 (multiple choice). Is the reason for probation or suspension always included in the MSPE/Dean's Letter?	Yes/No/Unsure
14 (multiple choice). Does your institution always include a record of remediation of failed coursework in the MSPE/Dean's Letter?	Yes/No/Unsure
15 (specify). If they answered no or unsure to *Q14, they were asked to please specify:	Answers varied to include the independent practices of some schools
16 (multiple choice). Is the reason for remediation of coursework always included in the MSPE/Dean's Letter?	Yes/No/Unsure
17 (Likert scale). Compared to other factors that are of significance in the residency match (e.g. USMLE scores, grades, research, letters of recommendation, personal statement, etc), how likely do you believe it is that successful remediation of each of the following disciplinary actions (taken individually) impacts a student's choice of medical specialty to apply to for residency? (17a) Failed Course (17b) Academic Probation (17c) Professionalism Probation (17d) Suspension	(1) Not at all likely (2) Somewhat likely (3) Moderately likely (4) Very likely (5) Extremely likely Unsure
18 (Likert scale). As in the previous question, compared to other factors that are of significance in the residency match, how likely do you believe it is that successful remediation of each of the following disciplinary actions (taken individually) impacts a residency program director's decision to interview the student? (18a) Failed Course (18b) Academic Probation (18c) Professionalism Probation (18d) Suspension	(1) Not at all likely (2) Somewhat likely (3) Moderately likely (4) Very likely (5) Extremely likely Unsure
19 (Likert scale). As in the previous question, compared to other factors that are of significance in the residency match, how likely do believe it is that successful remediation of each of the following disciplinary actions (taken individually) impacts a student's risk of failing to match into a residency program? (19a) Failed Course (19b) Academic Probation (19c) Professionalism Probation (19d) Suspension	(1) Not at all likely (2) Somewhat likely (3) Moderately likely (4) Very likely (5) Extremely likely Unsure
20 (multiple choice). Is your medical school public or private?	Public/Private
21 (multiple choice). Average class size per year at your medical school?	51 to 100 Students, 101 to 150 Students, 151 to 200 Students, 201 to 250 Students, Greater than 250 Students
22 (multiple choice). Number of years you have served as a dean?	1 to 5 years, 6 to 10 years, 11 to 15 years, 16 to 20 years, 26 to 30 years
23 (written values). Your age?	Answers ranged from 39 to 70-years-old
24 (multiple choice). Gender?	Male/Female

**Table 4:** Survey questions for residency program directors MSPE: Medical Student Performance Evaluation

QUESTION	ANSWER CHOICES
1 (Likert scale). How much variability in the number of students put on probation or suspension as disciplinary actions do you believe exists at different U.S. medical schools?	(1) Not at all variable (2) Slightly variable (3) Moderately variable (4) Very variable (5) Extremely variable
2 (Likert scale). How much variability in the criteria to put students on probation or suspension as disciplinary actions do you believe exists at different U.S. medical schools?	(1) Not at all variable (2) Slightly variable (3) Moderately variable (4) Very variable (5) Extremely variable
3 (percentage value). What percentage of U.S. medical schools do you believe consistently report their students' complete history of probation or suspension in the MSPE/Dean's Letter?	0 to 100%
4 (percentage value). What percentage of U.S. medical schools do you believe consistently report their students' complete history of remediation of failed coursework (in the absence of probation or suspension) in the MSPE/Dean's Letter?	0 to 100%
5 (Likert scale). Compared to other factors that are of significance in the residency match (e.g. USMLE scores, grades, research, letters of recommendation, personal statement, etc), how likely do you believe it is that successful remediation of each of the following disciplinary actions (taken individually) impacts a residency program director's decision to interview the student? (5a) Failed Course (5b) Academic Probation (5c) Professionalism Probation (5d) Suspension	(1) Not at all likely (2) Slightly likely (3) Moderately likely (4) Very likely (5) Extremely likely
6 (Likert scale). As in the previous question, compared to other factors that are of significance in the residency match, how likely do believe it is that successful remediation of each of the following disciplinary actions (taken individually) impacts a student's risk of failing to match into a residency program? (5a) Failed Course (5b) Academic Probation (5c) Professionalism Probation (5d) Suspension	(1) Not at all likely (2) Slightly likely (3) Moderately likely (4) Very likely (5) Extremely likely
7 (multiple choice). Medical specialty?	Obstetrics/Gynecology, Surgery, Neurology, Internal Medicine, Pediatrics, Family Medicine, Radiology, Other
8 (multiple choice). Number of years you have served as a residency program director?	0 years, 1 to 5 years, 6 to 10 years, 11 to 15 years, 16 to 20 years, 21 to 25 years, 26 to 30 years, Greater than 30 years
9 (entered value). Your age?	Answers ranging from 35 to 76-years-old
10 (multiple choice). Your gender?	Female/Male
